# Comparative Study of NAP-XPS and Cryo-XPS for the Investigation of Surface Chemistry of the Bacterial Cell-Envelope

**DOI:** 10.3389/fchem.2021.666161

**Published:** 2021-04-30

**Authors:** Marit Kjærvik, Madeleine Ramstedt, Karin Schwibbert, Paul M. Dietrich, Wolfgang E. S. Unger

**Affiliations:** ^1^ Surface Analysis and Interfacial Chemistry, Bundesanstalt für Materialforschung und -prüfung (BAM), Berlin, Germany; ^2^ Department of Chemistry, Umeå University, Umeå, Sweden; ^3^ SPECS Surface Nano Analysis GmbH, Berlin, Germany

**Keywords:** cryo XPS, bacteria, *Pseudomonas fluorescens*, DSM 50090, hydrated samples, reference sample, C 1s spectra, near ambient pressure XPS

## Abstract

Bacteria generally interact with the environment via processes involving their cell-envelope. Thus, techniques that may shed light on their surface chemistry are attractive tools for providing an understanding of bacterial interactions. One of these tools is Al Kα-excited photoelectron spectroscopy (XPS) with its estimated information depth of <10 nm. XPS-analyses of bacteria have been performed for several decades on freeze-dried specimens in order to be compatible with the vacuum in the analysis chamber of the spectrometer. A limitation of these studies has been that the freeze-drying method may collapse cell structure as well as introduce surface contaminants. However, recent developments in XPS allow for analysis of biological samples at near ambient pressure (NAP-XPS) or as frozen hydrated specimens (cryo-XPS) in vacuum. In this work, we have analyzed bacterial samples from a reference strain of the Gram-negative bacterium *Pseudomonas fluorescens* using both techniques. We compare the results obtained and, in general, observe good agreement between the two techniques. Furthermore, we discuss advantages and disadvantages with the two analysis approaches and the output data they provide. XPS reference data from the bacterial strain are provided, and we propose that planktonic cells of this strain (DSM 50090) are used as a reference material for surface chemical analysis of bacterial systems.

## Introduction

Bacteria are ubiquitous in our environment and contribute to both health and disease depending on circumstances and context. Many bacterial processes are mediated through their cell envelope. Thus, tools that can provide a description of the surface chemistry of bacterial cells are of great interest for life sciences, especially analysis techniques and methods that can be applied onto intact cells with minimum pre-treatment. In this light, X-ray photoelectron spectroscopy (XPS), in its different applications, is an interesting tool as it provides the chemical composition and binding states of atoms at the near surface of a sample. Al Kα has been one of the most common monochromatic X-ray sources in commercial instruments enabling higher energy resolution compared to non-monochromatic beams, e.g., emitted by Mg Kα anodes. When using Al Kα X-rays for excitation of photoelectrons the information depth is <10 nm. Historically, XPS analyses of biological samples were performed on freeze-dried specimens to allow compatibility with the ultrahigh-vacuum system of the spectrometer (van der Mei et al., 2000; Rouxhet et al., 2008; Rouxhet and Genet, 2011). Rouxhet et al. performed detailed work on how to perform this process with a minimum of carbon contamination during sample preparation by freeze drying (Rouxhet et al., 1994).

Today, applications of XPS exist that allow for analyses of hydrated samples without introducing a drying step (Ramstedt et al., 2011; Kjaervik et al., 2018; Hagberg et al., 2020; Shchukarev et al., 2020). One alternative is cryo-XPS where the sample is fast frozen, a treatment that vitrifies the water in the cells conserving their spatial structure. Running cryo-XPS, these frozen samples are analyzed at liquid nitrogen temperature under ultra-high vacuum (UHV) conditions in the analysis chamber of the XPS instrument. Another development is near-ambient pressure XPS (NAP-XPS) that makes it possible to characterize samples in various gas atmospheres, including water vapor, at pressures in the mbar-range and the need for compatibility of a sample to UHV conditions is by-passed. These techniques provide increased flexibility with respect to sample preparation since biological samples can remain hydrated.

### Near-Ambient Pressure NAP-XPS

The earliest development of NAP-XPS happened already in the 60 and 70s by Hans and Kai Siegbahn, the first XPS-spectra of liquids were from formamide (Siegbahn and Siegbahn, 1973). They used various methods to introduce the liquid into the measurement chamber, e.g., a liquid jet, spinning disk, by moving a fine wire through a liquid reservoir or a wetted rotating-wheel system (Siegbahn, 1985). In the 1990 and 2000s, advancements in differentially pumped electrostatic lenses led to an increase in maximum pressure in which samples could be measured (Ogletree et al., 2002). Nowadays, NAP-XPS instruments are widely available at synchrotron facilities and commercial instruments for laboratory-use are on the market (Arble et al., 2018). For this work, the laboratory NAP-XPS instrument EnviroESCA (SPECS Surface Nano Analysis GmbH, Germany) was used. It is equipped with a three-stage differential pumping system, a PHOIBOS NAP 150 electron energy analyzer and a monochromated Al Kα X-ray source. In the used experimental setup, NAP conditions were realized by backfilling the measurement chamber with water vapor (further details are given in *Materials and Methods*).

### Cryo-XPS

Cryo-XPS relies on a sample fast-freezing technique first developed to study processes at the water - mineral interface (Ramstedt et al., 2002; Shchukarev et al., 2004, 2007, 2020; Shchukarev and Ramstedt, 2017) and later applied to biological systems (Leone et al., 2006; Ramstedt et al., 2011, Ramstedt et al.,2014; Shchukarev et al., 2020). By rapidly freezing a hydrated sample, amorphous water is maintained at the solid surface of a material. Thus, the methodology is believed to enable analysis of intact hydrated microbial cells without damaging, reorganizing and/or rupturing cell membranes (Ramstedt et al., 2014; Shchukarev and Ramstedt, 2017). Generally, the sample is introduced at room temperature and fast-frozen in the loading chamber of the spectrometer under a flow of dry nitrogen gas before the pressure is reduced and the sample transferred to the analysis chamber for analysis at liquid nitrogen temperature. This procedure leads to evaporation of some bulk water from the surface of the sample. However, a few layers of water remain covering the solid material (Shchukarev et al., 2007) in addition to water present inside the sample structure. For biological samples, this relates to structural water at the surface as well as inside cells, and thereby enables analysis of intact cells without cell collapse. For mineral surfaces, this frozen water cover was estimated to represent 2-3 layers of water molecules. This was based on measured differences in intensity for main elements between frozen samples and samples re-measured in the same spot after water evaporation (Shchukarev and Sjöberg, 2005). Thus, the cryo-XPS methodology allows for probing the solid surface through the water layers remaining at the solid-water interface. Furthermore, the formation of this hydrated surface, in combination with the evaporation of bulk water during sample freezing and transfer, has been shown to give rise to very low levels of adventitious carbon at the surface of mineral samples. On the surface of frozen drops of aqueous solutions, it was not detected at all (Shchukarev et al., 2004, 2007; Shchukarev and Ramstedt, 2017). Previous studies have shown that radiation damage and thermal decomposition of samples under the X-ray beam can be prevented for redox sensitive compounds by performing the analysis under liquid nitrogen temperatures (Chidambaram et al., 2001). Thus, XPS analyses at liquid-nitrogen temperatures have broadly been hypothesized to reduce the problem of thermal-induced beam damage for a variety of samples including biological samples, as observed for other X-ray techniques (Snell et al., 2007).

### XPS Analyses of Bacterial Cells

**FIGURE 1:**
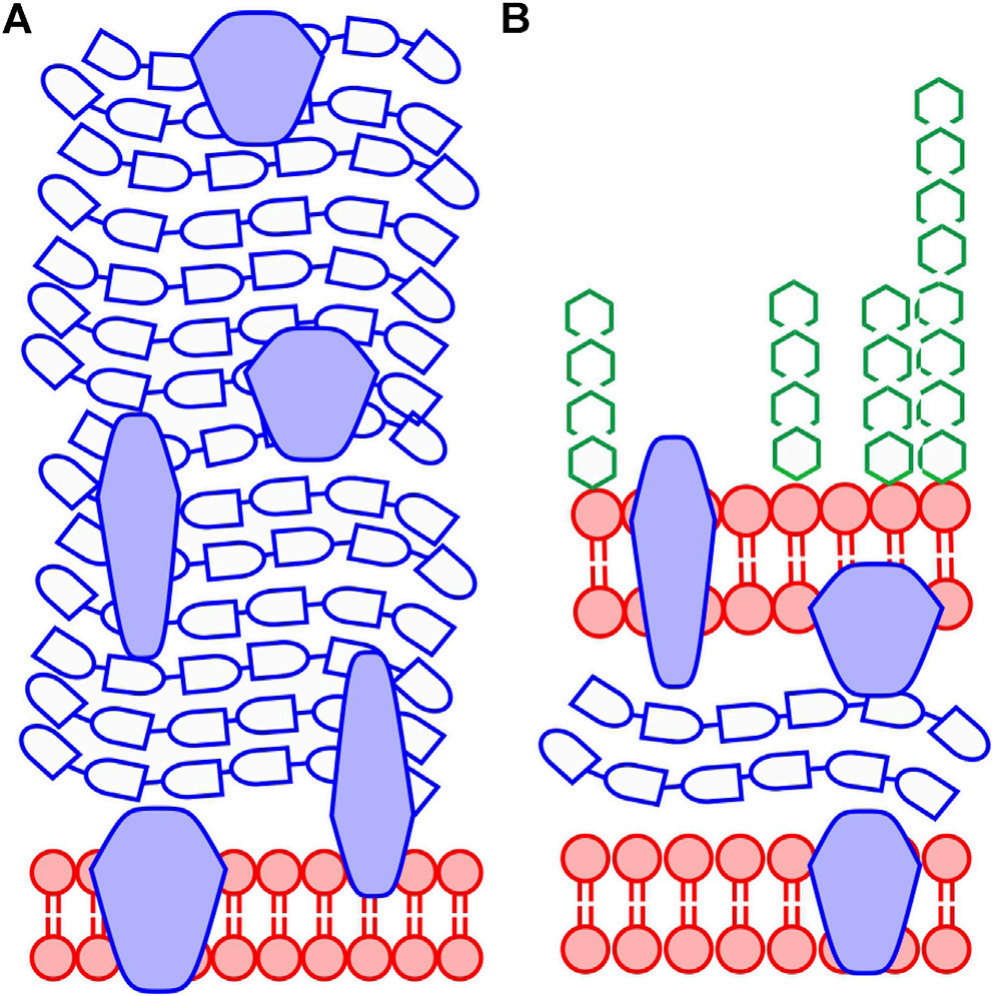
Generalized schematic structure of the cell envelope for bacteria. **(A)** Gram-positive bacteria have a thick peptidoglycan layer covering the plasma membrane **(B)** Gram-negative bacteria have a cell envelope consisting of an inner plasma membrane, a thin peptidoglycan layer, and an asymmetric outer membrane consisting of lipids and lipopolysaccharides. Throughout the cell envelope, there are proteins with various functions for both types of bacteria. The color coding of the figure relates to the substance groups discussed in this work: lipid-like substances (red), peptide containing substances, i.e., protein and peptidoglycan (blue) and polysaccharides (green). Proteins and other structures may be glycosylated and in the Gram-positive cell envelope there are teichoic acids and lipoteichoic acids containing polysaccharide-like structures (not shown in the figure). The *Pseudomonas fluorescens* bacteria analyzed here are Gram-negative.

Due to the information depth of XPS, only the outermost structures of the bacterial cell envelope will be analyzed, and the chemical composition is expected to vary depending on bacterial species. The outermost part of the cell envelope of Gram-negative bacteria consists of an asymmetric lipopolysaccharide membrane, called the outer membrane, which has a variety of proteins embedded. Beneath this membrane is a volume called the periplasm with a thin peptidoglycan layer that gives stability to the cell envelope (Figure 1B). The inner membrane separates the cytoplasm of the cell from the periplasmic space (Silhavy et al., 2010). The thickness of the cell envelope has been reported to be in the range 10–40 nm, thus, thicker than the information depth of XPS. Previous studies have indicated that XPS probes only the outer membrane and possibly a part of the thin peptidoglycan layer in the periplasmic space in Gram-negative bacteria (Ramstedt et al., 2011). For Gram-positive bacteria, the outermost part of the cell consists of a thick peptidoglycan layer (30–100 nm) with embedded proteins, teichoic acids, and lipoteichoic acids (Silhavy et al., 2010; Ramstedt et al., 2014) (Figure 1A). Thus, XPS will probe only the outermost part of the peptidoglycan layer in the cell envelope of Gram-positive bacteria. However, it is important to remember that if the bacterial surface is covered by surface structures, such as capsule, flagella, or pili, these will contribute to the spectra. Depending on their volume, they may also influence how “deep” into the cell envelope information is obtained during analysis. As an example, XPS analyses of bacterial cells with a thick capsule would likely not give information about the cell envelope but mainly describe the chemistry of the capsule surrounding the cell.

Bacterial systems have been analyzed by XPS for more than three decades. Extensive work has been performed to quantify the elemental composition of the surface of freeze-dried bacteria, as illustrated in the review by van der Mei et al. (van der Mei et al., 2000). Initial work focused on ratios between elements as well as data treatment using equation systems to deduce the substance composition with respect to the amount of the three main organic building blocks of the bacterial cell-envelope: lipids/hydrocarbon-like compounds, polysaccharides, and proteins/peptides (Figure 1). (Rouxhet et al., 1994). Rouxhet et al. also developed a second equation series for determining substance composition from the C1s spectrum, alone, using peak fitting with Gaussian-Lorentzian mixed peak shapes (Rouxhet and Genet, 2011). The first of these two equation series, relates the content of major macromolecular substance groups in the cell wall to their contributions in O, N, and C spectra. The second relates the content of these substance groups to their contribution to the C1s spectrum. Thus, both can be seen as an advanced form of quantification based on mass balance. A similar approach was performed by Ramstedt et al. (Ramstedt et al., 2011), applying multivariate curve resolution to a collection of bacterial samples and standard substances to obtain spectral components that predicted the surface content of lipids, protein/peptidoglycan and polysaccharide from the C1s spectra of bacteria (Ramstedt et al., 2011) here called “the Umeå method”. Comparisons between the “Umeå method” with the equation systems developed by Rouxhet et al. have shown that they give comparable, albeit not identical, results. They both relate the XPS data to the composition of “ideal substances”, but the spectral fingerprints of these ideal substances are not identical (Hagberg et al., 2020).

As water is present at the surface of hydrated samples, a complete elemental quantification only relating to the bacterial cell envelope is neither obtained in cryo-XPS nor in NAP-XPS. Secondly, for NAP-XPS, the photoelectrons are attenuated by inelastic scattering events in the surrounding gas, depending on their kinetic energy and the nature of the gas. Thus, for NAP-XPS quantification is only possible when the attenuation of the signal is accounted for (Khalifa et al., 2018; Tao and Nguyen, 2018; Jürgensen et al., 2019).

For both cryo-XPS and NAP-XPS-based methods, Rouxhet’s first equation system (Rouxhet et al., 1994; Rouxhet and Genet, 2011) correlating elemental composition to surface content is not applicable. Thus, in our aim to benchmark the two methods NAP-XPS and cryo-XPS against one another, we have chosen to use a data treatment method focusing on predicting substance composition only from peak fitting analysis of high-resolution C1s spectra from a reference strain of *Pseudomonas fluorescens* (DSM50090). This reference strain is available from the German culture collection.^1^ We here provide reference data for XPS analyses of this Gram-negative bacterium in planktonic form, and we suggest this strain is suitable as a reference strain enabling comparisons between different studies and laboratories.

## Materials and Methods

*P. fluorescens* (Strain DSM50090^1^) were grown on Luria Broth^2^ (LB) agar plates at room temperature for 48 h. For NAP-XPS analysis, bacteria were taken from the agar plate with a sterile spatula and gently spread on a Si-wafer. A video illustrating the sample preparation is available in the supplementary material. For cryo-XPS analysis, bacteria were taken from the culture plate using a sterile inoculation loop, placed on the sample holder, fast-frozen and analyzed directly. Alternatively, the bacterial cells were washed with PBS (containing 8 g L^−1^ NaCl, 0.2 g L^−1^ KCl, 1.44 g L^−1^ Na_2_HPO_4_, 0.24 g L^−1^ KH_2_PO_4_ at pH 7.4), centrifuged, the supernatant discarded, and approximately 20 µL of the pellet was transferred using an automatic pipette to the sample holder forming a dense droplet (this wash was performed only for some of the replica of cryo-XPS, and not for NAP-XPS). Thereafter, the bacterial biomass was fast-frozen in the sample introduction chamber under a gentle flow of dry nitrogen, the pressure reduced, the sample transferred into the analysis chamber, and analyzed by cryo-XPS in accordance with previously described procedures (Ramstedt and Shchukarev, 2016; Hagberg et al., 2020). Previous studies have shown no significant differences in major peaks (C1s and N1s) between washed and non-washed *P. fluorescens* bacteria grown on LB agar culture plates (Hagberg et al., 2020).

Cryo-XPS measurements were performed on an Axis Ultra DLD electron spectrometer (Kratos Analytical Ltd., Manchester, United Kingdom) at liquid nitrogen temperature (−160°C). A monochromatic Al Kα source was operated at 150 W and an analyzer pass energy of 160 or 20 eV was used for survey spectra and high-resolution spectra, respectively. Sample charging was compensated by the built-in spectrometer charge-neutralizing system and the binding energy of the aliphatic carbon C1s component peak was referenced to 285.0 eV. Peak fitting analysis of the C1s peaks was done by using CasaXPS with Gaussian-Lorentzian peak shape models, as well as using the Umeå-method predicting the contribution in percent of C atoms by fitting the C1s spectra with spectral components related to lipid, peptide (proteins and peptidoglycan) and polysaccharide (Ramstedt et al., 2011; Ramstedt and Shchukarev, 2016; Hagberg et al., 2020).

NAP-XPS measurements were performed with an EnviroESCA instrument (SPECS Surface Nano Analysis GmbH, Berlin, Germany), equipped with a monochromatic Al Kα X-ray source and a PHOIBOS 150 NAP electron energy analyzer, see ref. (Dietrich et al., 2019). for a more detailed instrumental description. The sample was inserted to the sample introduction chamber immediately after the bacteria had been applied to the Si-wafer. The chamber was pumped down, and at approximately 400 mbar water vapor was dosed into the analysis chamber by a manual leak valve connected to a sealed glass vial containing degassed water (ultrapure HPLC grade, Alfa Aesar). This way, the sample was never exposed to high or ultra-high vacuum. A video illustrating this is available in the supplementary material. At approximately 20 mbar, the valve between the introduction chamber and analysis chamber was opened. The gas composition was checked by monitoring the O1s and N1s spectra in survey-scans. When nitrogen was no longer detected, the sample was put into measurement position.

**FIGURE 2:**
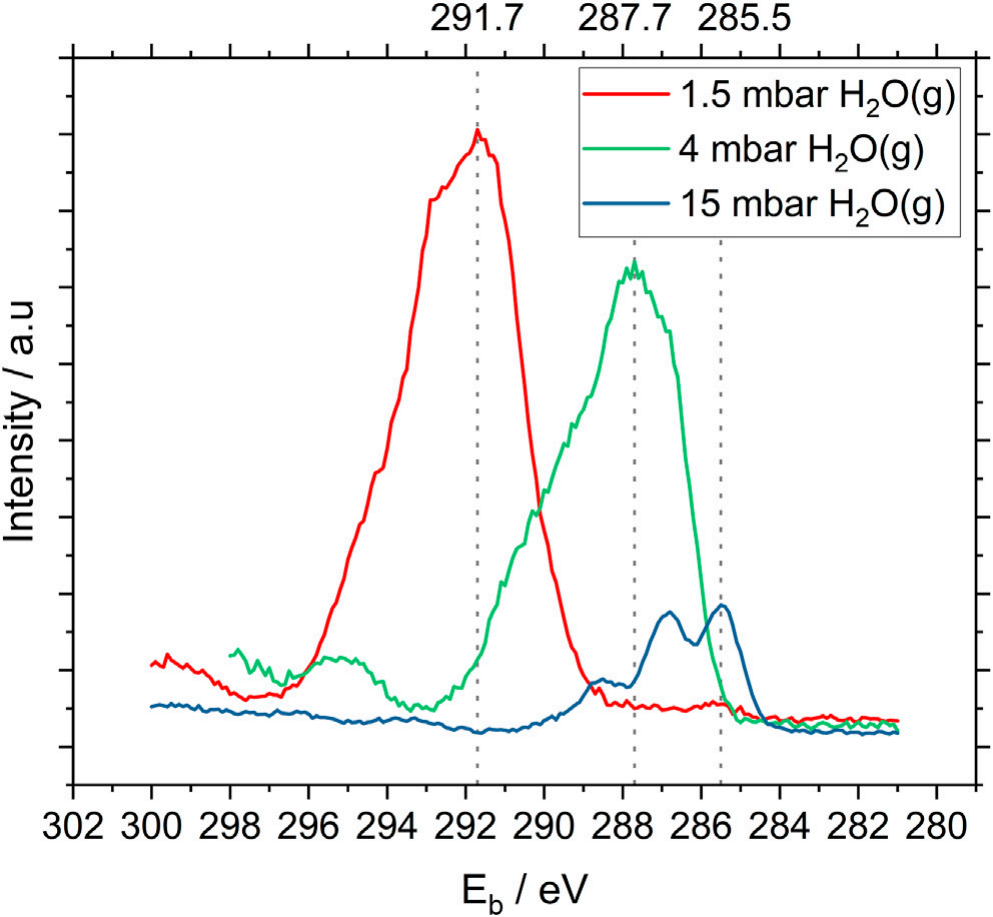
High resolution C1s NAP-XP -spectra of *P. fluorescens* acquired under different (increasing) pressures of water vapor in the analysis chamber of the instrument. Count rate maxima are indicated by the stippled lines. Note that these spectra are not corrected for surface charging.

To establish an optimal measurement protocol for NAP-XPS analyses, spectra were acquired at various pressures of water vapor. Figure 2 illustrates the relationship between water vapor pressure, count rates measured for C1s photoelectrons and surface charging measured with the bacteria sample. At 1.5 mbar of water vapor, the maximum C1s count rate was 4.3 times higher than at 15 mbar. However, due to charging effects, the spectrum was shifted 6.7 eV towards higher binding energy and the spectral resolution was poor. At 15 mbar, the spectrum was shifted only 0.5 eV towards higher binding energy and the component peaks in the C1s spectrum can clearly be distinguished. These conditions, delivering high resolution in binding energy, were chosen for comparison of NAP-XPS results with data from cryo-XPS. The count rate was inherently low but had an acceptable level of noise. Therefore, all the NAP-XPS spectra (except Figure 2) shown here were acquired under 15 mbar humidity in the analysis chamber of the instrument. Full spectrum acquisition parameters are listed in Supplementary Table S1.

The photoelectron signal is attenuated in NAP-conditions due to scattering of electrons by the gas phase, which depends on the kinetic energy of the electrons, type of gas, and pressure. The electron attenuation in water vapor was investigated following the same procedure as Khalifa et al. (2017), using the well-characterized ionic liquid, [C3C1im][NTf2] (Holzweber et al., 2019). This gave attenuation factors for carbon and nitrogen. As proposed by Tao et al. (2018), these were used to correct the atomic ratios of C and N in NAP-conditions. Quantifying the magnitude of the electron attenuation is still work in progress and will enable correct quantitative surface chemical analysis for all relevant elements by a determination of a transmission function (Holzweber et al., 2019) in NAP-XPS at a given ambient pressure in the future.

### Data Analysis

Unifit 2020^3^ or CasaXPS^4^ were used for peak fitting analysis. In Unifit 2020, component peaks were fitted with the program’s Lorentzian-Gaussian sum-function to model the peak shape. The binding energy scale was corrected for surface charging with respect to the C1s component peak of aliphatic carbon at 285.0 eV, in accordance with ISO 19318:2004. The peak fitting parameters and constraints for NAP-XPS analysis are listed in Supplementary Table S2. Generally, the Lorentzian-Gaussian mixing factor was constrained to be the same for all components within the same spectrum. An exception for this is the gas component from water vapor in the O1s spectrum for NAP-XPS, which inherently has a different peak shape and a narrower FWHM than the component peaks from the sample. Further, the binding energy of component 4 in the C1s spectrum was constrained to be 4.1 eV higher than that of component 1 for spectra from NAP-XPS, corresponding to the binding energy shift for carbon in carboxyl groups of the lipids and proteins (Rouxhet and Genet, 2011). The background was fitted with a Shirley-background.

The fitting procedure in CasaXPS was as follows: The background was fitted using a Shirley background defining the area of the peak. This area was thereafter fitted with as few components as possible and using Gaussian-Lorentzian, GL30, peak shapes, but without further restrictions (if not stated otherwise). The binding energy was corrected for surface charging with respect to the C1s component peak of aliphatic carbon at 285.0 eV (ISO 19318:2004) after the peak fitting procedure. Thereafter, a second analysis of the C1s spectra was performed by fitting the spectra using the Umeå method, as previously described (Ramstedt et al., 2011; Ramstedt and Shchukarev, 2016). In short, this method relies on three spectral components that predict the contribution of main constituents of the bacterial cell-envelope, i.e., carbohydrates, lipids and protein/peptidoglycan, to the C1s peak. Thus, within a surface layer characterized by the information depth of the XPS method. C1s spectra with calibrated binding energy scale were fitted with the three spectral components representing the three different substance classes and the relative area of these components in the C1s spectrum predicted their relative carbon atom percentages.

## Results

### NAP-XPS

**FIGURE 3:**
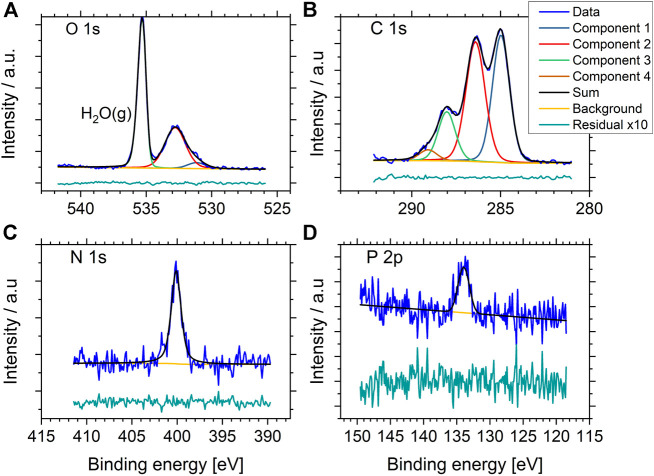
Representative high resolution O1s, C1s, N1s, and P2p NAP- XP spectra of *P. fluorescens* held in 15 mbar water vapor atmosphere during analysis. The green tracks display the residuals after subtracting the sum of the component peaks from the experimental spectrum (blue), multiplied by 10 for better visibility.

A total of three samples of *P. fluorescens* were analyzed, two areas of one of the samples and three areas of another sample, resulting in a total of six datasets. From this, we obtained reproducible data, both between different samples and different areas of the same sample (Supplementary Figure S1 shows C1s spectra of all replica, Supplementary Figure S2 shows wide spectra). Representative high-resolution NAP XP-spectra of *P. fluorescens* are shown in Figure 3. Well resolved spectra were obtained from C1s, O1s, and N1s. In addition, minor contributions from P2p were detected. Since PBS, or other buffers, were not used during sample preparation, this signal originated from the bacterial cell envelope. Overall, the spectra obtained were similar to published data from *E. coli* in NAP-conditions (Kjærvik et al., 2018), freeze-dried bacteria in regular XPS, and fast-frozen bacteria in cryo-XPS (Rouxhet et al., 2011; Ramstedt et al., 2011; Ramstedt et al., 2014; Ramstedt and Shchukarev 2016).

The high-resolution spectrum of O1s in Figure 3A gives a visual impression of the gas signal vs. the sample signal, the maximum count rate for the gas phase line is 3.7 times higher than the maximum count rate from the sample-signal. Since the O1s gas peak is well separated in binding energy to the O1s peak from the sample, the signal can be excluded from quantitative analysis and does not affect the quantitative results directly. However, due to the presence of water in the samples, the measured amount of oxygen cannot be directly correlated to the surface chemistry of the bacterial cell-envelope.

For comparison with data obtained from cryo-XPS, we therefore focused on the C1s-line and the component distribution gained from 1) peak fitting analysis of C1s and 2) the percentage of lipids, polysaccharides and protein/peptidoglycan obtained using the Umeå-method. The C1s spectrum was fitted with four components. The ratio relative to total carbon content was 0.37±0.01 aliphatic carbon at binding energy 285.0 eV, 0.43±0.01 carbon with O or N as nearest neighbor at 286.4 eV, 0.16±0.01 carbon in peptide bond at 288.1 eV and 0.04±0.01 carbon in carboxyl groups at 289.1 eV. Further details on carbon ratios, FWHM and binding energy are listed in Supplementary Table S2. Using the “Umeå-method”, the surface composition was predicted to 57±6% of C atoms in protein/peptidoglycan, 17±4% of C atoms in lipid, and 26±2% of C atoms in polysaccharide (Supplementary Table S4).

### Cryo-XPS

Analysis of nine samples of the *P. fluorescens* reference strain using cryo-XPS, yielded highly reproducible data (Supplementary Tables S3, S4 and Supplementary Figure S3). The average surface elemental composition was 58±2 at% C, 10±1 at% N and 29±2 at% O (including water). Furthermore, minor elements were present such as S (approx. 0.1 at%) and elements that may originate both from the bacterial cell envelope and PBS, i.e., Na, K, Cl, and P.

The nine samples analyzed using cryo-XPS contained both cells that were washed with PBS and cells analyzed directly from culture plates. The four samples analyzed directly from plates displayed a composition for the minor elements of 0.5±0.06 at% Na, 0.4±0.03 at% Cl, and 0.4±0.01 at% P. All these minor elements were present at higher content in the five washed samples indicating slight contribution from the PBS buffer: 1.5±0.6 at% Na, 0.2±0.04 at% K, 1.0±0.4 at% Cl, and 0.8±0.1 at% P. No difference was observed for C and N between washed and not washed samples, indicating that this reference strain was not producing extracellular substances when it was grown on LB agar. Such substances would have been removed during the washing procedure and given rise to differences between washed and not washed samples (Hagberg et al., 2020).

The carbon peak was fitted with four components (GL30) and resulted in a distribution in relation to total C of: 36.5±1.1% aliphatic carbon at 285.0 eV; 41.9±1.2% carbon with an O or N neighbor at 286.5 eV; 18.9±1.2% C in a peptide bond at 288.1 eV; as well as 2.6±1.3% C in carboxyl groups at 289.1±0.2 eV. The binding energies were in accordance with previous reports (Leone et al., 2006; Rouxhet and Genet, 2011). The nitrogen was fitted with two components that represented non-protonated nitrogen in amine and amide groups (400.1 eV) as well as a smaller contribution of protonated N (401.6± 0.1 eV) in amine or amide groups at the surface, as previously reported for other bacterial strains (Leone et al., 2006; Rouxhet and Genet, 2011). Detection of protonated amine groups has previously been shown for hydrated samples analyzed with cryo-XPS. After sample drying, this protonation was lost due to rearrangement of the proton position between zwitterionic groups (Ramstedt et al., 2004). The oxygen peak was fitted with two peaks at 532.9 and 531.5 eV where the former contained water. Both these peaks were assigned to have (some) contributions from O in the bacterial cell envelope. A low content of S could be detected at the surface of the bacterial cells, ca 0.1 at%. It was assigned to thiols at 163.7±0.2 eV (Castner et al., 1996). Four replica with longer acquisition time also contained traces of sulfonate at 168.9±0.4 eV probably indicating rearrangement of the water layer coverage at analysis times longer than 1 h, altering the information depth at the site of analysis. However, future work is needed to test that hypothesis. No sulfonate was detected at the surface of samples exposed to X-rays for less than 1 h under liquid nitrogen cooling (Supplementary Figures S3–S5).

In order to predict the contribution of peptides (proteins and peptidoglycans), lipids and polysaccharides in the C 1s spectra of the nine samples, “the Umeå method” was employed. It predicted that 66±4% of detected surface C atoms originated from proteins or peptidoglycans, 8±2% from lipids and 26±2 from polysaccharides (Supplementary Table S4), in line with what has previously been reported for bacterial cells (Hagberg et al., 2020; Ramstedt et al., 2011; Ramstedt et al., 2014).

### Comparison NAP-XPS and Cryo-XPS

**FIGURE 4:**
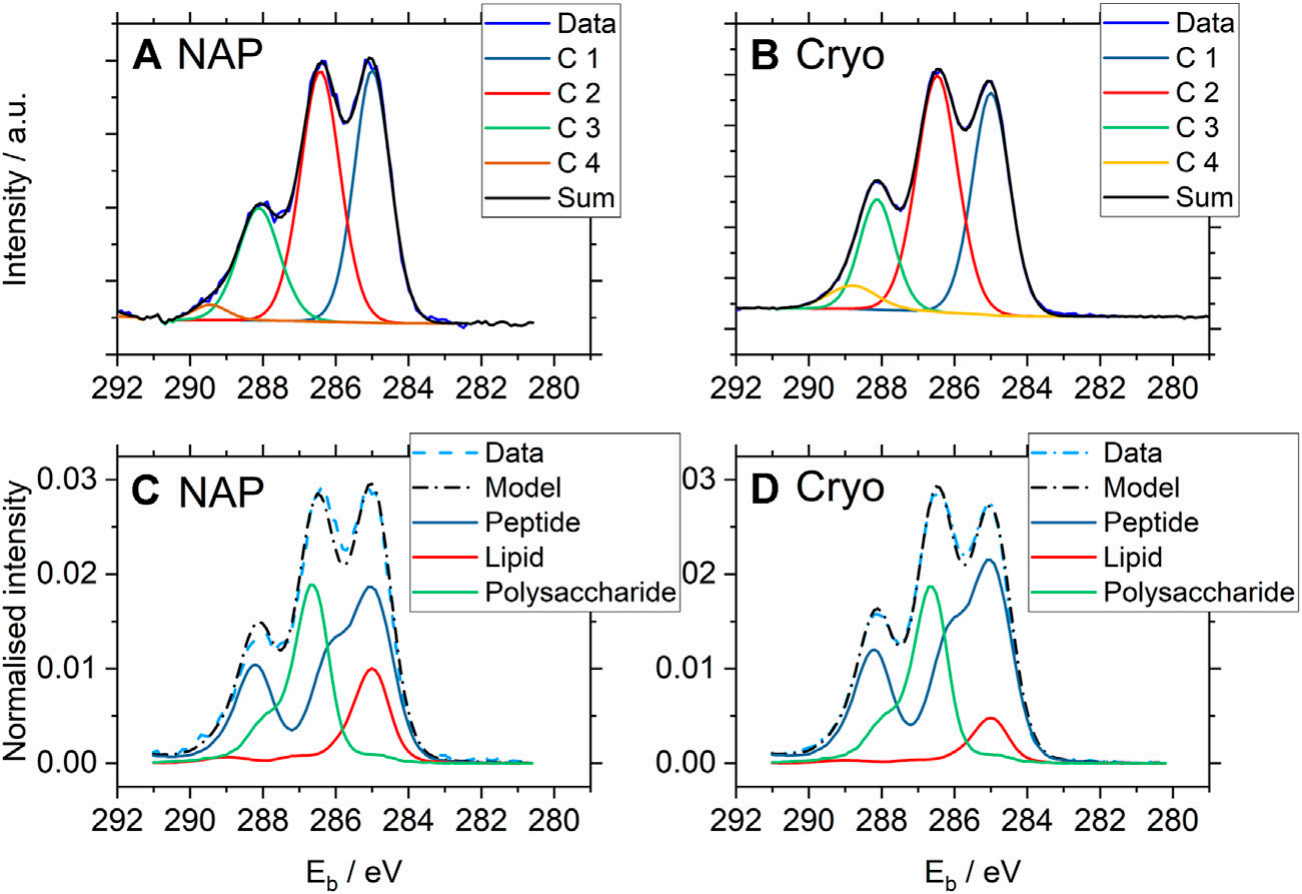
One selected representative high resolution C1s spectrum from each set of the NAP- and cryo-XP spectra of *P. fluorescens* acquired under NAP **(A, C)** and cryo **(B, D)** conditions. The binding energy scales were corrected for surface charging (i.e., the charging potential as defined in ISO 18115–1, term 4.103) with respect to the C1s component peak of aliphatic carbon at 285.0 eV, in accordance with ISO 19318:2004. The two upper spectra **(A, B)** were analyzed by peak fitting using CasaXPS with equal fitting strategy (for fitting parameters and results see Supplementary Tables S2–S4). C1 represents aliphatic carbon at binding energy 285.0 eV, C2 carbon in C-O or C-N bonds at 286.4 eV, C3 carbon in peptide bonds at 288.1 eV and C4 carbon in carboxyl groups at 289.1 eV. The two lower ones **(C, D)** show the results of spectral analysis using the Umeå method predicting the amount of substance related to the different molecular classes constituting the bacterial cell-envelope within a surface layer characterized by the information depth of the XPS method.

**FIGURE 5:**
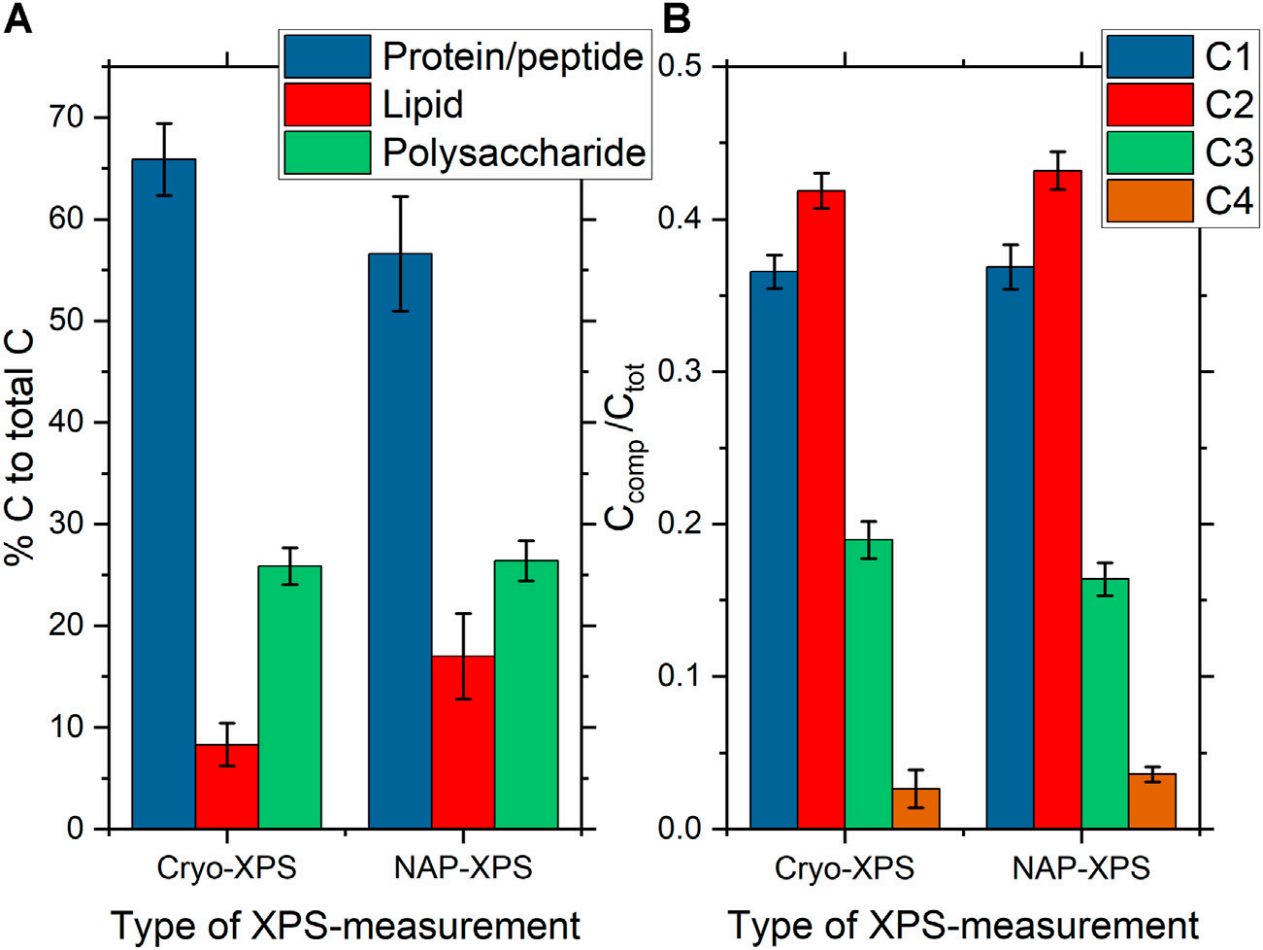
**(A)** Bar charts showing the average surface composition of *P. fluorescens* samples expressed as the amount of substance related to the different molecular classes constituting the bacterial cell-envelope measured under cryo-conditions (n = 9) and NAP-conditions (n = 6) as obtained by analysis of the respective C1s spectra using the Umeå method. Error bars represent the standard deviation. The percentage on the y-axis gives the predicted carbon atom percentages for the different molecular classes in the respective C1s spectra. **(B)** Bar charts showing the average C1s component peak intensities of *P. fluorescens* samples measured under cryo-conditions (n = 9) and NAP-conditions (n = 6) as obtained by analysis of the respective C1s spectra using peak fitting analysis of high resolution C1s with the CasaXPS software for cryo-XPS data and Unifit for NAP-XPS data. C1s component peaks are assigned in the caption to Figure 4.

C1s spectra from *P. fluorescens* were analyzed both by peak fitting analysis and using the Umeå method and results are displayed in Figures 4 and 5 (Please note that Figure 4 shows two individual C 1s spectra whereas Figure 5 reports averages of the entire dataset with standard deviations.). Data from the two techniques are in good agreement with each other, with three clearly distinguishable components (C1, C2, C3) and one smaller peak (C4). The *average* relative contributions (based on component peak areas) with the respective standard deviations of the four carbon-components for NAP- and cryo-XPS are displayed in Figure 5B (NAP-spectra were fitted with Unifit 2020 and cryo-spectra with CasaXPS). The relative contributions of three of these components (C1, C2, C4) to the C1s spectra are overlapping within the standard deviation. A small but statistically significant difference was found for component C3 (*p* < 0.01). Additionally, the resulting binding energies (BE) are in good agreement within 0.1 eV (Supplementary Tables S2, S3).

Furthermore, a similar content was observed for the three spectral components, protein/peptidoglycan, lipid, and polysaccharide, using the “Umeå-method” (Figure 5A, Supplementary Table S4). No significant differences in the surface composition were obtained between samples measured at NAP and cryo-conditions for protein/peptidoglycan and polysaccharide. For the lipids, a difference was observed with a higher percentage of lipid-like content obtained for NAP-XPS. Both protein/peptidoglycan and lipid give intensity in the C1s spectrum at 285.0 eV. Thus, the difference observed using the “Umeå-method” between NAP- and cryo-XPS relates to the difference observed in intensity at 288.1 eV, assigned to C in peptide bonds and is in agreement with differences in the N/C ratio obtained between the two methods. The N/C ratio in NAP-conditions after correction for electron attenuation in water vapor was estimated to 0.10, while it was 0.09 before correction. This small difference before and after correction is reasonable considering that C1s and N1s photoelectrons have similar kinetic energies (115 eV difference only). The N/C ratio was lower in NAP-conditions compared to cryo-conditions (0.10 vs 0.18). This correlated to the differences in component C3 in the C 1s-spectrum, which was also lower in NAP-conditions.

As the intensity of the signal is higher in cryo-XPS than in NAP-XPS, a minor second component in the N1s spectra could be resolved from the noise in N 1s spectra from cryo-XPS (Supplementary Figure S3). This smaller contribution has previously been assigned to protonated species at the surface (Leone et al., 2006). The higher signal intensity also allowed for detection of very small quantities of S with cryo-XPS. However, to obtain good signal to noise ratios long acquisition times were required, which appear to have given rise to some alterations of the surface composition within the probed surface volume. Apart from this observation, we did not detect any changes in surface chemistry vs. time of X-ray exposure. Thus, we conclude that neither in NAP-XPS nor cryo-XPS, the samples seem to suffer from radiation damage within the analysis periods used.

## Discussion

The comparison of data between NAP-XPS and cryo-XPS show overall good agreement. However, a higher content of lipid was observed in NAP-XPS (Figure 5, Supplementary Table S4). This is hypothesized to be a consequence of differences in the level of adventitious carbon depositing on the sample during analyses. This adventitious carbon would overlap with the signal from the lipid membrane and thereby give rise to apparent lipid concentrations that are higher than expected.

We conclude that both methods are suitable for these types of analyses and give comparable data. They have their distinct advantages and disadvantages. An advantage of cryo-XPS is its ultra-high vacuum provides a controlled environment with minimal surface contamination. Another advantage is the higher signal intensity obtained. In NAP-conditions, the signal intensity is always (inherently) lower caused by scattering losses in the gas atmosphere, thus, the minor elements (Na, Cl, P, K) are either at, or under the detection limit. With ongoing developments in NAP-XPS signal intensity, comparisons of minor elements will likely be possible in the future. However, for major elements, especially carbon, we have here shown that we obtain comparable data for cryo- and NAP-XPS. An advantage of doing measurements in NAP-conditions is that the time from sample insertion to data acquisition is short (approximately 30 min in this study). Four samples (each 1 × 1cm) can easily be mounted at the sample plate, thus several samples can be measured without sample transfer. Further, measurements are not restricted to solid (or frozen) samples, and the pressure and type of gas can be varied. Thus, higher levels of flexibility and other types of experimental designs may be possible with NAP-XPS.

### Sample Preparation and Surface Contamination

Surface contamination could originate from both contamination during sample pre-treatment as well as contamination from the atmosphere in and around the XPS equipment.

Analyses of fast-frozen hydrated bacterial cells using cryo-XPS will always include small quantities of the solution that the cells were suspended in before centrifugation. In order to rid this solution of components from the growth medium in which bacteria were cultivated, a washing step is generally applied. The composition of this wash solution should ensure the integrity of the cell and, ideally, not influence protonation of surface functional groups at the bacterial cell envelope. Thus, in order to maintain constant pH and salinity, a PBS buffer is generally chosen for the washing step. However, as any constituent in the wash solution may remain in the small volume of liquid surrounding cells, this procedure may introduce errors in the elemental composition of Na, K, Cl, and P. Consequently, in the dataset from cryo-XPS, presented here, we observe slightly higher content of these minor elements in washed samples compared to samples taken directly from a LB agar plate. However, washing did not influence the C and N peaks. This illustrates that for bacterial cells that do not secrete large amounts of extracellular substances when grown on culture plates, it is preferable to perform XPS analyses without a washing step, as was done for all the NAP-XPS analyses reported here. For bacterial strains that do secrete significant amounts of extracellular substances, a washing step will be required (Hagberg et al., 2020). The washing solution should not contain any substances carrying C atoms as they may give rise to intensity in the C1s spectrum. Furthermore, it is important to remember that any evaporation of solution may concentrate dissolved substances. Thus, if relatively large volumes of liquid are evaporated, the concentration of non-volatile solutes will increase and may affect the bacterial cells. Evaporation may also produce precipitation of salts covering the surface and change pH in a non-buffered system. As the solution content is very low in the centrifuged cell pellets used in cryo-XPS, these effects should not have given rise to this type of artifacts in this study. However, this indicates that applying the sample directly from the culture plate is preferable, when possible.

Surface contaminations from adventitious C in the atmosphere in or around the XPS instrument would be very difficult to distinguish from the lipid content of the bacterial cell and, thus, not possible to directly quantify. However, based on the previous experience of very low contamination levels for other types of hydrated samples during cryo-XPS, we hypothesize that this contribution is very low, also when it comes to analysis of bacterial cells. The bacterial cell remains hydrated in the same way as mineral surfaces do during the entire process. Thus, we postulate that the thin water layer that is formed creates a hydrophilic surface that is reducing adsorption of adventitious carbon at the surface of the sample. Possibly, this is why the lipid content appears to be higher in NAP-XPS than in cryo-XPS.

### *P. fluorescens* as Reference Strain

The bacterial reference strain (DSM50090), used in this study, is an environmental *P. fluorescens* strain that grows well at room temperature and can be grown beneficially on regular LB agar growth plates over a time period of several days. The strain is classified as non-pathogenic and, thus, can be handled in small quantities in regular labs (Bio-safety level BSL-1, with proper sterilization techniques used to avoid contamination). Therefore, this cultivation procedure can be seen as a comparably robust procedure to enable bacteria to remain viable and intact for longer periods of time (days) compared to bacteria in liquid broth. In contrast, bacteria growing in liquid broth will start to degrade after having reached the stationary phase when nutrients become limited. Furthermore, growth of bacteria in liquid broth requires a washing step to remove substances from the medium that may otherwise contribute to the spectra. Previous studies have shown that the reference strain, used here, secretes very low levels of extracellular substances when grown on LB agar. Thus, spectra of washed cells from liquid broth and cells from LB agar plates were not significantly different (Hagberg et al., 2020). This similarity means that bacterial biomass can be taken directly from the culture plate and transferred to the sample holder using a sterile culture loop. This gives a minimum of sample preparation, and thereby removes procedures that may give rise to contaminations and variations in sample surface composition.

We have here shown that the specific strain we have analyzed and compared (DSM50090) gives reproducible data between two laboratories, when analyzed directly from LB culture plates. Similar surface composition was obtained from the C1s peak, using two different XPS methods, two different programs for peak fit analysis, as well as two different persons performing the peak fitting. Thus, we suggest this bacterial strain as a reference material for surface chemical analysis. This could enable inter- and intra-laboratory comparisons of performance both with respect to the XPS techniques but also enabling XPS to be more easily compared to other surface sensitive analyses techniques (XPS spectra presented here are available as data sets in the online open-access repository Zenodo).

## Conclusion

*P. fluorescens* bacteria in planktonic form were successfully analyzed using NAP-XPS and cryo-XPS. The two methods allow for analysis of the hydrated bacterial cell-envelope of intact bacterial cells. Thus, they both circumvent the need for freeze-drying the bacteria and thereby reduce sample pretreatment and connected surface contamination. The data for the two methods nicely compare within the error bars, except for a higher surface content of lipid-like C at the surface of bacteria analyzed with NAP-XPS when evaluated by the Umeå method. We hypothesize that this originates from surface contamination of adventitious carbon in NAP-XPS that is very low in cryo-XPS compared to other XPS applications. Thus, the small discrepancy may originate from the fact that fast-frozen bacteria were immersed in a fast-frozen “protective” hydrated layer during cryo-XPS and, thus, less prone to surface contamination by aliphatic carbon species.

Furthermore, we suggest that the *P. fluorescens* strain DSM50090 is suitable as a live reference material for XPS analyses investigating the surface chemistry of the bacterial cell envelope.

## Data Availability

The datasets generated for this study can be found in the online open-access repository Zenodo with the DOI: 10.5281/zenodo.4525854.

## Footnotes

1
https://www.dsmz.de/collection/catalogue/details/culture/DSM-50090)

2
https://asm.org/Protocols/Luria-Broth-LB-and-Luria-Agar-LA-Media-and-Their-U

3
http://home.uni-leipzig.de/unifit/index.html

4
http://www.casaxps.com/
