# Supplementary material for: Comparative Study of NAP-XPS and Cryo-XPS for the Investigation of Surface Chemistry of the Bacterial Cell-Envelope
Source: Front Chem. 2021 Apr 30;9:666161. doi: 10.3389/fchem.2021.666161 (PMC8132101; doi:10.3389/fchem.2021.666161)
Supplement: Supplementary file 1 [file DataSheet1.pdf]

Supplementary Material

1 Supplementary Figures and Tables

1.1 Supplementary Figures

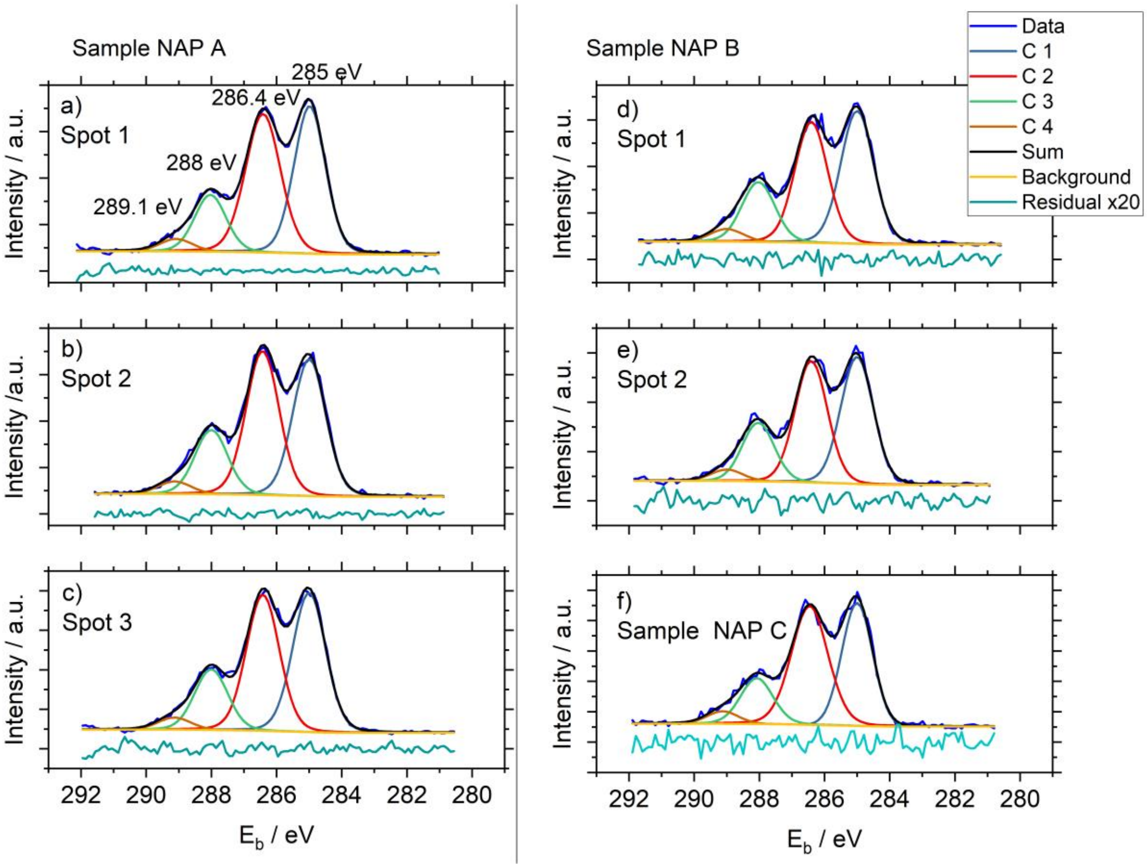

Figure S1: Fitted high resolution NAP XP C1s-spectra of two samples of *P. fluorescens*. Three spots were measured from sample NAP A (a,b,c), two spots were measured from sample NAP B (d,e) and one spot from sample NAP C.

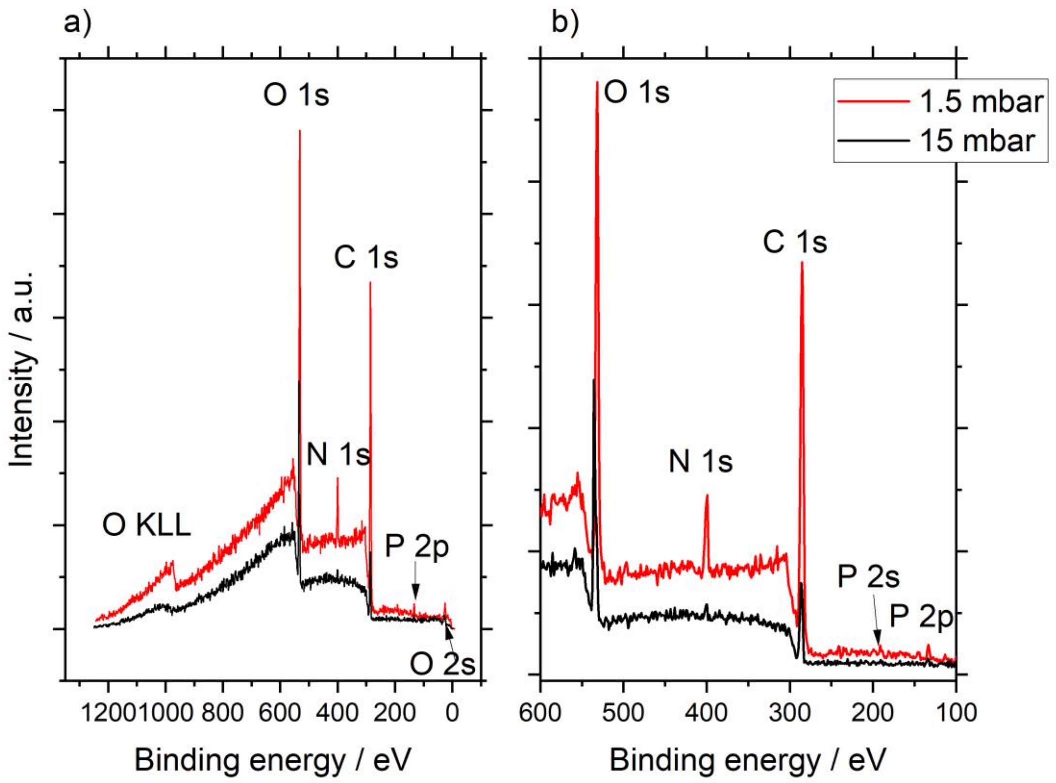

Figure S2: NAP-XPS survey spectra of *P. fluorescens* in 1.5 mbar and 15 mbar water vapor. Figure a) shows the whole energy range, while figure b) shows the energy range 100 to 600 eV.

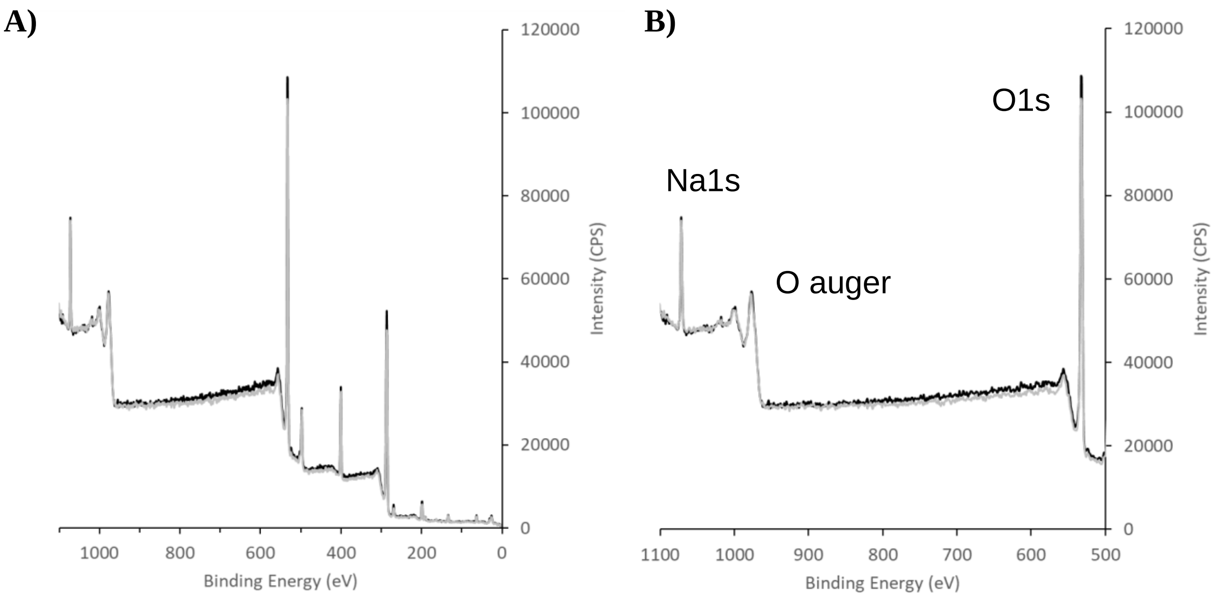

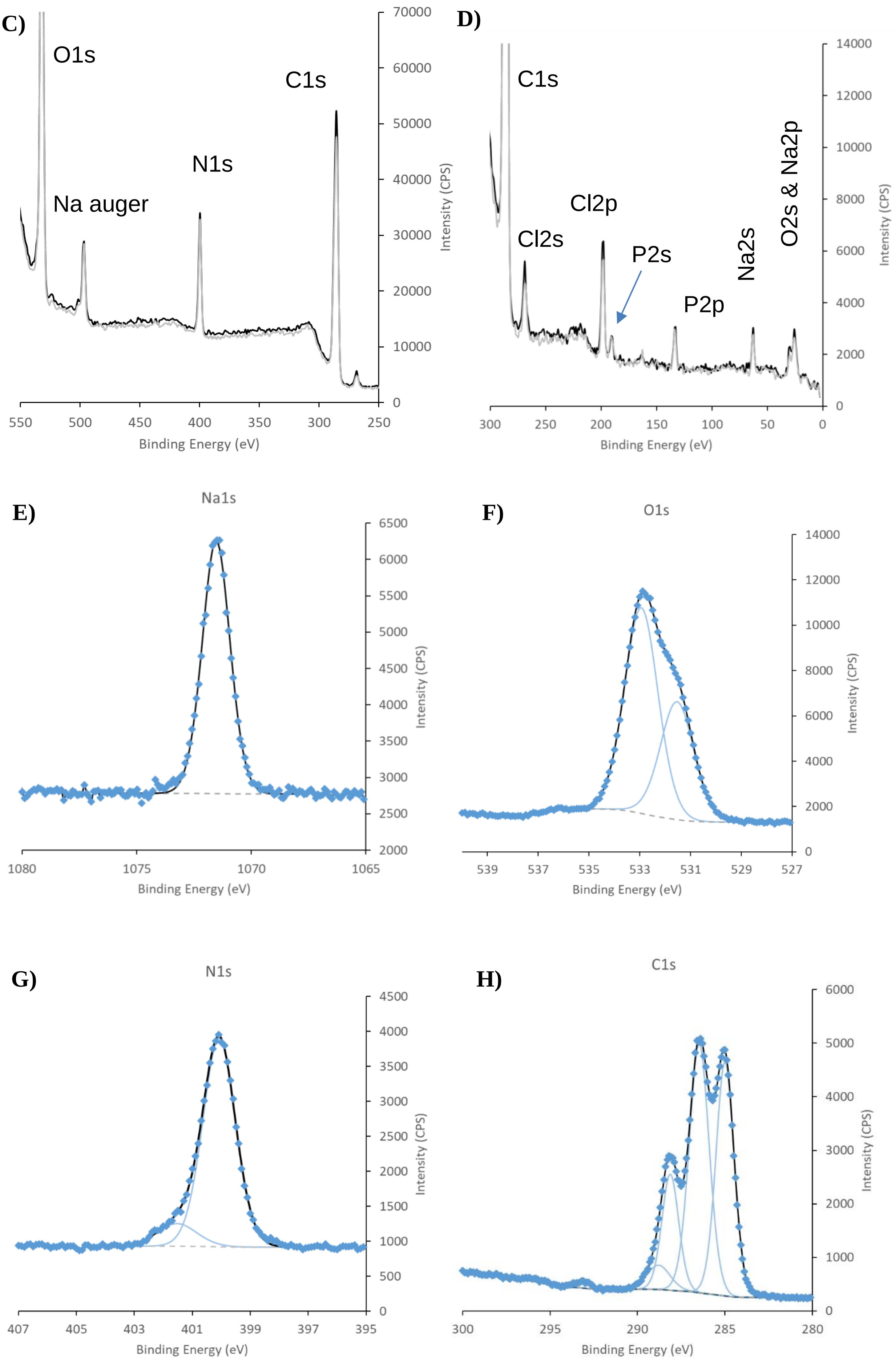
C)
O1s
Na auger
N1s
C1s
Intensity (CPS)
Binding Energy (eV)
D)
C1s
Cl2s
Cl2p
P2s
P2p
Na2s
O2s & Na2p
Intensity (CPS)
Binding Energy (eV)
E)
Na1s
Intensity (CPS)
Binding Energy (eV)
F)
O1s
Intensity (CPS)
Binding Energy (eV)
G)
N1s
Intensity (CPS)
Binding Energy (eV)
H)
C1s
Intensity (CPS)
Binding Energy (eV)

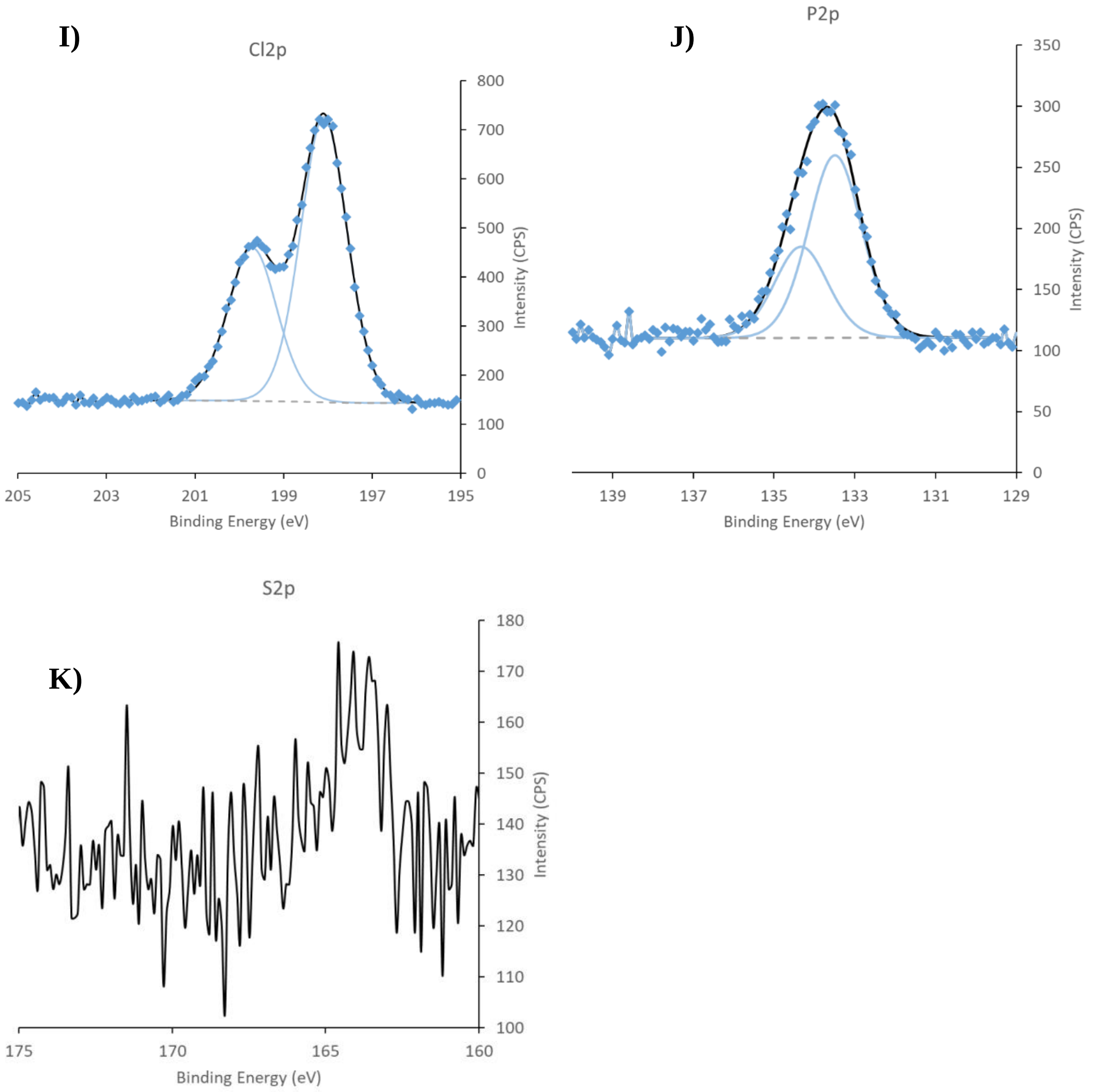

Figure S3: a) Cryo-XPS survey spectrum before (black line) and after analysis (grey line, acquisition time 3 min corresponding to 3 sweeps of 60s). b-d) enlargements of the survey spectra shown in a with labels for main lines. e-k) High resolution spectra of *P. fluorescens* from cryo-XPS (same replica as in Figure 4, acquisition time in parenthesis) washed with PBS; e) Na1s (3 min), f) O1s (3 min), g) N1s (5 min), h) C1s (7 min), i) Cl2p (7 min), j) P2p (7 min, fitted with linked $2p_{1/2}$ and $2p_{3/2}$), k) S2p (3 min). Non-fitted spectra (survey spectra and S2p) show data points as black lines. Fitted spectra show data points as blue diamonds, envelope as black line, Shirley background as grey broken line and fitted peak shapes in blue. Total acquisition time was ca 51 min.

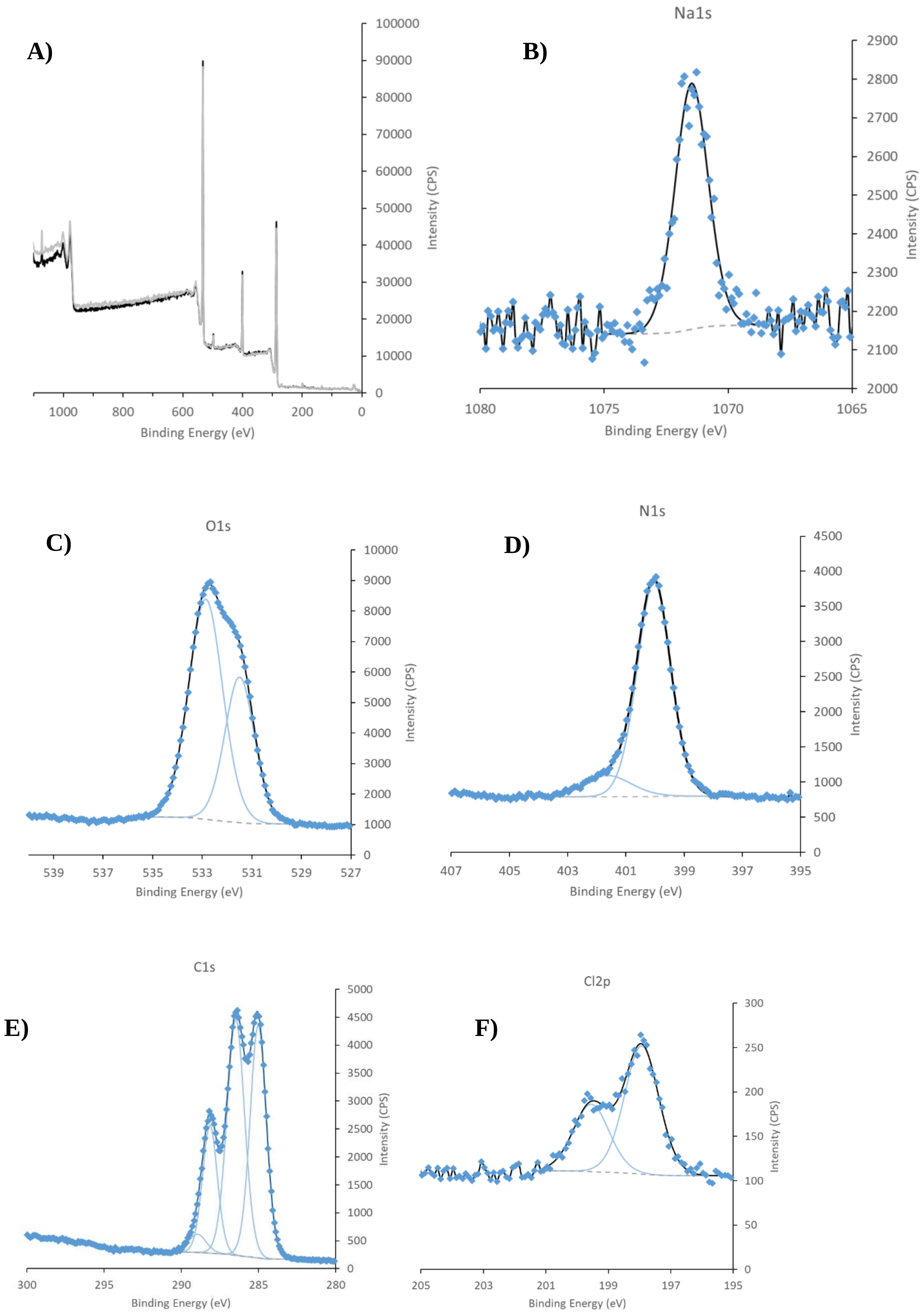
A)
Intensity (CPS)
Binding Energy (eV)
B)
Na1s
Intensity (CPS)
Binding Energy (eV)
C)
O1s
Intensity (CPS)
Binding Energy (eV)
D)
N1s
Intensity (CPS)
Binding Energy (eV)
E)
C1s
Intensity (CPS)
Binding Energy (eV)
F)
Cl2p
Intensity (CPS)
Binding Energy (eV)

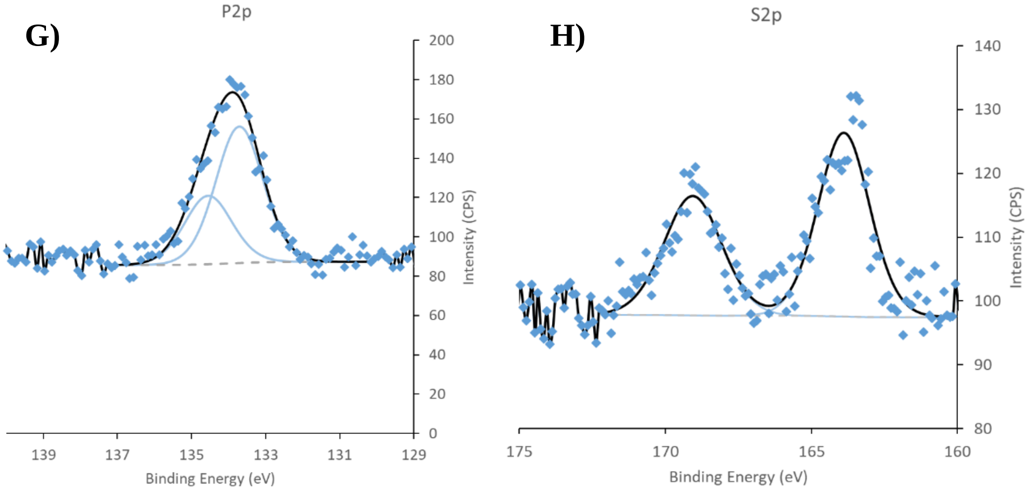

Figure S4: a) Cryo-XPS survey spectrum before (black line) and after analysis (grey line, acquisition time 3 min) for replica not washed with PBS. b-h) High resolution spectra of *P. fluorescens* from cryo-XPS (acquisition time in parenthesis); b) Na1s (3 min), c) O1s (3 min), d) N1s (5 min), e) C1s (7 min), f) Cl2p (7 min), g) P2p (7 min, fitted with linked $2p_{1/2}$ and $2p_{3/2}$), h) S2p (30 min). Non-fitted spectra (survey spectra and S2p) show data points as black lines. Fitted spectra show data points as blue diamonds, envelope as black line, Shirley background as grey broken line and fitted peak shapes in blue. Please note that the S2p doublet was fitted as a single peak only to get an approximate content and binding energy, due to its low intensity. Total acquisition time was ca 1h, 23 min.

A)

B)

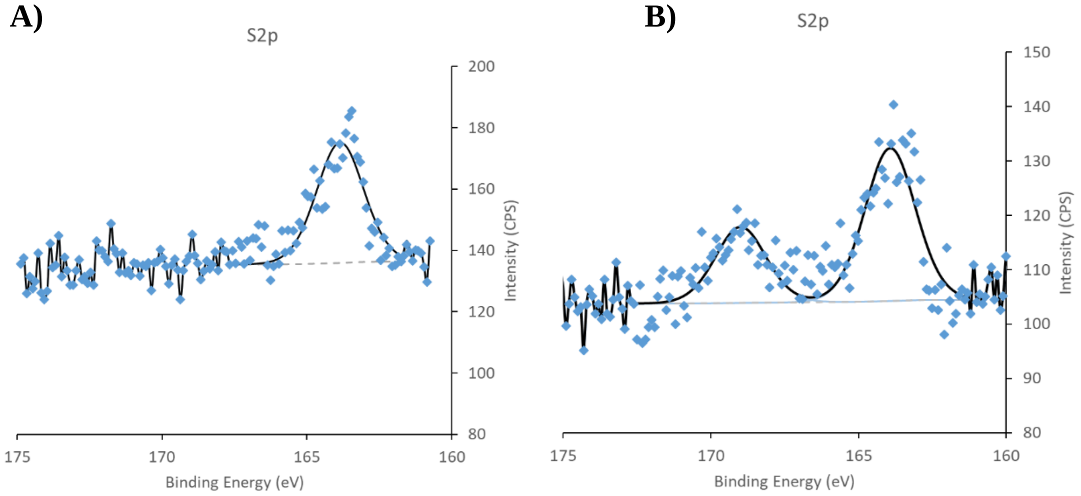

Figure S5: Cryo-XPS S2p spectrum from replica with total analysis time of a) 1h (acquisition time for S2p was 10 min i.e. 10 x 60 s) and b) 1h 20 min (acquisition time for S2p was 20 min i.e. 20 x 60 s whereas total acquisition time was influenced by acquisition time for all regions). S2p was fitted as one peak due to the noise level of spectra. Spectra show data points as blue diamonds, envelope as black line, background as grey broken line and fitted peak shapes in blue (coincides with the envelope of the fit, and in B overlaps also with the background of the other peak).

1.2 Supplementary Tables

Table S1: Acquisition parameters for NAP-XPS measurements.

XP-Spectra	No. of scans	Pass energy [eV]	Step size [eV]	Dwell time [s]	Acquisition time (mm:ss)*
Survey	2	100	1	0.1	4:15
C 1s	12	30	0.1	0.25	12:33
O 1s	5	30	0.1	0.25	6:42
N 1s	12	30	0.1	0.25	17:33
P 2p	12	30	0.1	0.25	12:22

*differs as the BE regions are not identical between elements

Table S2: Fit of bacterial spectra from NAP-XPS using a Shirley background and Unifit 2020, with standard dev in parenthesis. * indicates that the L-G parameter was constrained to the value of the first component. L-G mixing = Lorentzian-Gaussian mixing factor, LG=1 means a pure Lorentzian function. ** indicates that the binding energy difference between the first and the fourth component peak was constrained to be 4.1 eV[1].

NAP-XPS n=6	C1s				N1s	O1s		
Component	1	2	3	4	1	1	2	3 (gas phase)
Position Binding Energy (eV)	285.0 (0.0), ref.	286.4 (0.0)	288.1 (0.0)	289.1** (0.0)	400.1 (0.0)	531.2 (0.0)	532.8 (0.1)	535.4 (0.0)
FWHM (eV)	1.1 (0.1)	1.3 (0.1)	1.2 (0.1)	1.2 (0.1)	1.3 (0.1)	1.2 (0.2)	1.9 (0.0)	0.7 (0.0)
LG-mixing	0.1	0.1*	0.1*	0.1*	0.2	0.2	0.2*	0.2
Total elemental composition (at%), not corrected for electron attenuation in water vapor	63.5 (3.5)				5.5 (2.4)	29.9 (3.4)		
Ratio to tot C	0.369 (0.014)	0.432 (0.012)	0.164 (0.011)	0.036 (0.005)	0.086 (0.037)	0.475 (0.080)		

[1] Beamson, G., and Briggs, D. (1992). High resolution XPS of organic polymers. *Sci. ESCA300 Database*.

Table S3: Fit of bacterial spectra from cryo-XPS using CasaXPS with Shirley background. Average of 9 biological replicas, with standard dev in parenthesis.

n=9	C1s				N1s		O1s	
Component	1	2	3	4	1	2	1	2
Position Binding energy (eV)	285.0	286.5 (0.0)	288.1 (0.0)	289.1 (0.2)	400.1 (0.0)	401.6 (0.1)	532.9 (0.0)	531.5 (0.0)
FWHM	1.2 (0.0)	1.4 (0.1)	1.2 (0.1)	1.3 (0.3)	1.4 (0.1)	1.6 (0.2)	1.6 (0.1)	1.4 (0.1)
Gaussian-Lorentzian peak shape	GL(30)	GL(30)	GL(30)	GL(30)	GL(30)	GL(30)	GL(30)	GL(30)
Component composition at the surface (at%)	21.3 (0.7)	24.4 (1.1)	11.0 (0.7)	1.5 (0.7)	9.1 (0.8)	1.1 (0.3)	20.0 (2.3)	8.9 (0.9)
Total elemental composition (at%)	58.3 (1.7)				10.2 (1.0)		29.0 (1.6)	
Ratio to total C	0.365 (0.011)	0.419 (0.012)	0.189 (0.012)	0.026 (0.013)		0.175 (0.016)		0.498 (0.039)

Table S4: Average composition (and standard deviation) of the bacterial cell-envelope as obtained by analysis of C1s using the Umeå method for cryo-XPS and NAP-XPS spectra. Predicted substance composition in percentage of total C, n is the number of replicates.

Method	n	Peptide	St. dev.	Lipid	St. dev	Polysaccharide	St. dev
Cryo-XPS	9	66	4	8	2	26	2
NAP-XPS	6	57	6	17	4	26	2
